# Dual-Function Femtosecond Laser: β-TCP Structuring and AgNP Synthesis via Photoreduction with Azorean Green Tea for Enhanced Osteointegration and Antibacterial Properties

**DOI:** 10.3390/ma17205057

**Published:** 2024-10-16

**Authors:** Marco Oliveira, Liliya Angelova, Liliana Grenho, Maria Helena Fernandes, Albena Daskalova

**Affiliations:** 1 Institute of Electronics, Bulgarian Academy of Sciences, 72 Tsarigradsko Chaussee Blvd, 1784 Sofia, Bulgaria; lily1986@abv.bg; 2 BoneLab—Laboratory for Bone Metabolism and Regeneration, Faculty of Dental Medicine, University of Porto, 4200-393 Porto, Portugal; lgrenho@fmd.up.pt (L.G.); mhfernandes@fmd.up.pt (M.H.F.); 3 LAQV/REQUIMTE, University of Porto, 4160-007 Porto, Portugal

**Keywords:** β-tricalcium phosphate, surface structuring, biocompatibility, femtosecond laser, silver nanoparticles, photoreduction, antibacterial potential

## Abstract

β-Tricalcium phosphate (β-TCP) is a well-established biomaterial for bone regeneration, highly regarded for its biocompatibility and osteoconductivity. However, its clinical efficacy is often compromised by susceptibility to bacterial infections. In this study, we address this limitation by integrating femtosecond (fs)-laser processing with the concurrent synthesis of silver nanoparticles (AgNPs) mediated by Azorean green tea leaf extract (GTLE), which is known for its rich antioxidant and anti-inflammatory properties. The fs laser was employed to modify the surface of β-TCP scaffolds by varying scanning velocities, fluences, and patterns. The resulting patterns, formed at lower scanning velocities, display organized nanostructures, along with enhanced roughness and wettability, as characterized by Scanning Electron Microscopy (SEM), optical profilometry, and contact angle measurements. Concurrently, the femtosecond laser facilitated the photoreduction of silver ions in the presence of GTLE, enabling the efficient synthesis of small, spherical AgNPs, as confirmed by UV–vis spectroscopy, Transmission Electron Microscopy (TEM), and Fourier Transform Infrared Spectroscopy (FTIR). The resulting AgNP-embedded β-TCP scaffolds exhibited a significantly improved cell viability and elongation of human bone marrow mesenchymal stem cells (hBM-MSCs), alongside significant antibacterial activity against *Staphylococcus aureus* (*S. aureus*). This study underscores the transformative potential of combining femtosecond laser surface modification with GTLE-mediated AgNP synthesis, presenting a novel and effective strategy for enhancing the performance of β-TCP scaffolds in bone-tissue engineering.

## 1. Introduction

Bone loss, whether resulting from trauma, tumor removal, infection, or congenital disorders, presents a significant challenge in orthopedic surgery, often leading to the delayed healing or non-union of fractures [1]. To address these complex bone defects, bone substitutes have become a critical solution. A bone substitute is a synthetic, inorganic, or biologically derived material designed to replace autologous or allogenic bone in repairing such defects [2]. Over the past 50 years, a variety of bone substitutes have been utilized in clinical practice, which can be broadly classified into three main categories: bone grafts, ceramics, and growth factors. Bone grafts include autografts (harvested from the patient’s own tissue), allografts (sourced from human donors), and xenografts (obtained from other species). Ceramics consist of materials like hydroxyapatite, tricalcium phosphate, and calcium sulfate, while growth factors include compounds such as demineralized bone matrix, platelet-rich plasma, and bone morphogenetic proteins [3].

While bone autografts have been the standard approach for promoting bone repair, their limitations, including limited availability and potential complications, necessitate the exploration of alternative materials [4,5].

β-TCP is a bioceramic material highly regarded for its biocompatibility and bioactivity in bone repair [6]. It has been shown to enhance bone regeneration more effectively than nanostructured carbon implants, porous titanium, and even bone autografts in animal studies [7,8]. To optimize bone healing, β-TCP scaffolds have been modified by adjusting physical characteristics such as pore size, porosity, surface roughness, and by incorporating ionic components or growth factors.

However, the same properties that make β-TCP biocompatible can also render it susceptible to bacterial infections, a leading cause of implant failure. These infections are particularly challenging, often necessitating implant replacement or, in severe cases, resulting in amputation or even death [9,10]. Bacteria adhere to implant surfaces, forming biofilms that lead to complicated treatment [10].

The interaction between cells and surfaces can be influenced by several factors such as chemical composition, surface energy, wettability, and topography [11,12,13]. Modifying the surface roughness at the micro or nanoscale can physically impede bacterial attachment [13]. Despite its advantages in tissue engineering, calcium phosphate-based scaffolds, including β-TCP, are not inherently bactericidal and can still be colonized by bacteria after implantation [13]. Functionalization strategies such as antibacterial peptide immobilization, antibiotic loading, or ion release are often employed to mitigate this risk, though the widespread use of antibiotics raises concerns about resistance [14].

Femtosecond (fs)-laser surface processing offers a promising method for preventing bacterial adhesion by creating a physical barrier on the material surface [15]. The interaction of ultra-short laser pulses develops with the minimization of thermal diffusion, thus avoiding unwanted damage to the material [16,17]. Studies have shown that fs-laser texturing can effectively reduce bacterial viability by altering surface topography. For example, Chen et al. demonstrated that nanorough microchannels on borosilicate glass reduced the viability of *Escherichia coli* and *S. aureus* through mechanical stress [18]. Similarly, Jalil et al. observed that variations in laser fluence and pulse width could disrupt bacterial adhesion on gold surfaces by creating disordered nanostructures [19].

However, due to the crystalline nature of β-TCP, laser structuring presents both significant antibacterial potential and challenges. While this treatment can create a physical barrier against bacterial colonization, maintaining a certain level of porosity makes the material susceptible to bacterial colonization [20].

In this context, AgNPs offer a promising approach when combined with β-TCP, thanks to their dual beneficial effects, including their antibacterial potential and stimulation of osteogenesis [21,22]. There are several methods available for synthesizing metallic nanoparticles, with chemical methods being the most commonly used. However, the use of toxic chemicals, such as reducing agents like sodium borohydride, N,N-dimethylformamide, and hydrazine [23,24], has led to the emergence of greener alternatives [25].

Nanoparticle synthesis using fs-laser technology has emerged as a transformative method in this field, eliminating the need for toxic chemicals and allowing for the precise production of nanoparticles for biomedical applications. In particular, Keshavarz et al. synthesized silica nanoparticles (SiNPs) via fs laser, which induced selective apoptosis in cancer cells [26]. Similarly, Zelepukin et al. demonstrated the safety and effective tumor accumulation of fs-laser-synthesized titanium nitride (TiN) nanoparticles, highlighting their potential in phototheranostic applications [27]. Nevertheless, most recent studies on the photoreduction of AgNPs in water require the use of scavengers to counteract rapid back oxidation by hydroxyl radicals. Compounds such as ammonium have been used as scavengers, but this chemical can also be toxic and has even been linked to tumorigenesis [28].

GTLE has been extensively used as a reducing/stabilizing agent in the production of AgNPs. The polyphenols in green tea facilitate the reduction of Ag^+^ to AgNPs. Additionally, polyphenols help stabilize the AgNPs by adsorbing onto their surface. These polyphenols (e.g., catechins) also possess antioxidant and anti-inflammatory properties, which have demonstrated osteoprotective and osteogenic effects [29]. However, producing AgNPs using only GTLE can take at least 2 h to achieve a considerable concentration [30,31].

In light of this, the present study has two main objectives: first, to modify the surface structure of β-TCP using fs-laser processing, and second, to synthesize AgNPs through fs-laser photoreduction, employing an extract from the leaves of the Azorean *Camellia sinensis* to enhance the reducing and stabilizing reaction environment. The aim is to develop a material that integrates these two technologies—β-TCP modified by fs-laser processing and AgNPs synthesized through fs-laser photoreduction, with GTLE as a reducing/stabilizing agent—resulting in a surface capable of forming a barrier against bacterial infections while simultaneously promoting the proliferation of hBM-MCs. The results demonstrate that a higher number of applied laser pulses (lower scanning velocities) during the ablation of β-TCP leads to enhanced nanoscale structural organization. Furthermore, the combination of fs-laser photoreduction with the reducing potential of GTLE significantly improves the efficiency of AgNP production, achieving small, spherical nanoparticles in less than 2 min. The deposition of these AgNPs on the laser-modified β-TCP surface notably increased cell proliferation and significantly reduced bacterial colonization.

The importance of this study is highlighted by its innovative approach to tackling critical issues in orthopedic applications, such as bacterial infection and bone regeneration. By combining fs-laser technology with a green-tea-assisted synthesis method, the study offers a novel strategy for developing advanced biomaterials that provide antibacterial properties and promote the proliferation of bone cells. This dual functionality has the potential to not only enhance the effectiveness of bone implants but also reduce the risk of post-surgical infections, potentially leading to improved clinical outcomes for patients.

## 2. Materials and Methods

### 2.1. Fabrication of β-TCP Scaffolds

**Figure 1:**
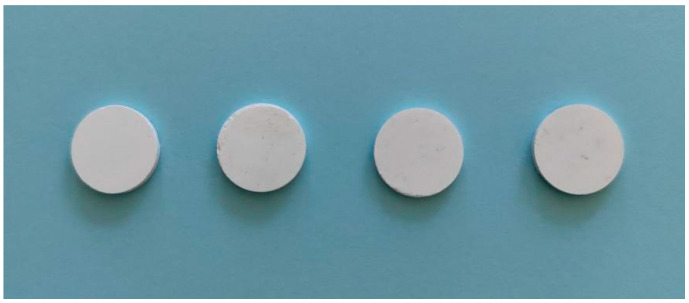
Representative image of four β-TCP samples prepared for fs-laser treatment.

To prepare the β-TCP disks, a co-precipitation technique was used. Initially, calcium nitrate tetrahydrate was dissolved in distilled water within a 6 L double-walled glass reactor. Ammonium phosphate dibasic was then introduced into the reactor using a peristaltic pump at a controlled rate of 10 mL/min. The reaction conditions were carefully maintained at 31 °C and a pH of 6.7, which was achieved by adding ammonia. The mixture was continuously stirred mechanically, and, after the addition of the ammonium phosphate dibasic solution was completed, it was left to mature for 20 h under the same stirring conditions. The resultant slurry was then filtered and dried. The dried material was subjected to a three-step calcination process and subsequently milled into a fine powder. This powder was then mixed with distilled water and Darvan C^®^ dispersant (Vanderbilt Minerals, Norwal, CT, USA) in a ball mill to produce a slurry. The slurry was poured into molds and allowed to dry overnight at 40 °C, followed by sintering to produce β-TCP disks with a diameter of 1 cm (Figure 1).

### 2.2. Laser-Processing of β-TCP Scaffolds

**Figure 2:**
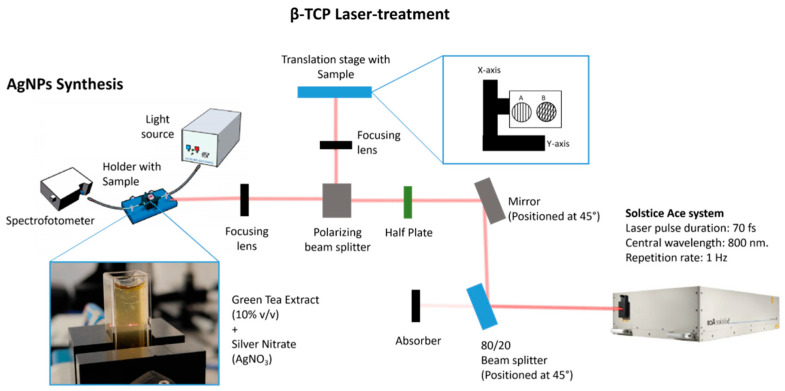
Schematic diagram illustrating the laser setup utilized for the surface treatment of β-TCP samples and the subsequent synthesis of AgNPs, each carried out as distinct steps.

Laser ablation was performed as shown in Figure 1, using a Solstice Ace system (Spectra-Physics, Milpitas, CA, USA) featuring a laser pulse duration of 70 femtoseconds and a central wavelength of 800 nm. The samples were affixed to a glass slide utilizing double-sided adhesive tape and positioned on a two-axis motorized translation stage (Thorlabs, Newton, NJ, USA) controlled via Kinesis^®^ Software v1.14.45 (Thorlabs, Newton, NJ, USA). The laser beam was directed through an 80/20 beam splitter, enabling 80% of the incident light to be utilized for experimental purposes, while 20% was allocated for routine diagnostics (e.g., the measurement of pulse width and beam profile). The beam was then reflected by an optical mirror to guide its path. Laser energy modulation was implemented using a polarization-based beam splitter in conjunction with a half-wave plate. The laser beam was focused onto the sample surface with an achromatic convex lens featuring a 200 mm focal length, resulting in a focal spot diameter of 25 μm (Figure 2). The ablation process was conducted at a constant repetition rate of 1 kHz for all samples. The disk surfaces were systematically raster scanned at varying scanning velocities (1, 2, 3.44, 5, 10, and 15 mm/s) and fluences (4.1 and 6.1 J/cm^2^). The ablation patterns, illustrated in Figure 2, consisted of either parallel lines (linear pattern) or grid-like formations (crossed pattern) with intersecting orthogonal lines at 45° angles. All modifications utilized a hatch distance of 50 µm, except for the grid patterns, where a hatch distance of 100 µm was used for the intersecting lines.

### 2.3. Morphological Characterization of the Laser-Treated β-TCP Scaffolds

#### 2.3.1. Scanning Electron Microscopy (SEM)

Laser-treated matrices were analyzed using SEM (“Lyra”, Tescan Orsay Holding, Brno-Kohoutovice, Czech Republic; TM4000 Hitachi High-Tech Europe). To enhance image clarity, a thin gold layer (~4 nm) was sputter-coated onto the samples before imaging. SEM imaging was performed with an accelerating voltage of 20 kV.

#### 2.3.2. 3D Optical Profilometry

Surface morphological changes induced by varying laser pulse applications were assessed using a 3D optical profilometry system (Leica DCM 3D, Berlin, Germany). The images were captured at 20× magnification in true color. The surface roughness of the modified regions was quantified according to ISO 4287 standard [32], specifically using the arithmetical mean height (Sa) as the measurement parameter. The 3D optical data were processed using ProfilmOnline software (www.profilmonline.com, accessed on 2 June 2024).

#### 2.3.3. Wettability Analysis

To evaluate the influence of the laser treatments in surface wettability, a DSA100 Drop Shape Analyzer (KRÜSS GmbH, Hamburg, Germany) was utilized, which is an optical system for video-based contact angle measurements. The wetting properties of both untreated and laser-modified surfaces were tested using distilled water (high polarity). Contact angles were measured at room temperature using the sessile drop technique with 2 μL droplets. A minimum of three droplets was used for each sample type. The evolution of each droplet was tracked for a total of three minutes, with measurements taken every second for the first minute and every subsequent minute. Contact angles were calculated using ADVANCE software v1.7.2.2 (KRÜSS, Hamburg, Germany), fitting the droplet profiles to the Young–Laplace equation.

### 2.4. Green Laser-Assisted Synthesis of AgNPs and Deposition in β-TCP Pellets

AgNPs were synthesized using silver nitrate as the precursor, GTLE as the stabilizing and reducing agent, and femtosecond laser irradiation. The key reduction reaction, where Ag^+^ is converted to AgNPs by the polyphenols in GTLE, can be represented as follows [33]:

(1) $$n{Ag}^{+}+\mathrm{Ar}-{\left(OH\right)}_{0}\to \;n{Ag}^{+}+\mathrm{nAr}=O+{nH}^{+}$$

where *Ar* denotes the phenyl group present in the polyphenols, and *n* indicates the number of hydroxyl groups oxidized by *Ag*^+^.

The extracts were prepared following the adaptation of the protocol described by [34], and 8 g of dried green tea leaves from Gorreana Tea Plantations, São Miguel-Azores, Portugal, was boiled in 50 mL of ultrapure water for 5 min. The solution was then cooled and filtered, and the resulting filtrate was stored at 4 °C as a stock solution. To initiate the synthesis, 400 µL of a 10 mM silver nitrate solution was mixed with 4 mL of a 10% (*v*/*v*) green tea extract solution. Immediately after the addition of the silver nitrate precursor, the mixture was subjected to laser irradiation at two different fluences (8.1 and 16.3 J/cm^2^) for 20 min using the Solstice Ace system, the same used for β-TCP treatment as represented in Figure 1. The same methodology was also applied without the addition of GTLE.

Following irradiation, the synthesized AgNPs were concentrated and purified by centrifugation at 4000 rpm for 10 min and then washed with deionized water to achieve a final volume of 4 mL.

After synthesis, the resulting AgNPs were deposited in the β-TCP scaffolds laser processed at a velocity of 1 mm/s and a laser fluence of 4.1 J/cm^2^. A concentration of 2 mM of the smallest AgNPs obtained was deposited and left to dry in ambient temperature for 8 h.

#### 2.4.1. UV–Vis Spectrometry

The real-time monitoring of the conversion of metal precursor to nanoparticles was conducted using an in situ UV–vis spectrophotometer (Ocean Optics USB2000, Orlando, FL, USA). Throughout laser processing, the absorption spectrum of the solution shifted from that of the metal precursor to that characteristic of the nanoparticle product. The laser irradiation ceased upon the stabilization of the absorbance at the specified wavelength, indicating the completion of nanoparticle formation (20 min).

#### 2.4.2. Fourier Transform Infrared (FTIR) Spectra Analysis

The FTIR spectra were registered in KBr ring pellets with a diameter of 13 mm. All spectra were recorded on a Nicolet Avatar 360 FTIR spectrometer (SpectraLab Scientific Inc., Markham, ON, Canada) at a spectral resolution of 2 cm^−1^ and an accumulation of 64 scans. The spectra were scanned in the 4000–400 cm^−1^ range. The treatment and analysis of the spectra were made using the OMNIC advanced software v9.14 (Thermo Fisher Scientific Inc., Waltham, MA, USA).

#### 2.4.3. Transmission Electron Microscopy (TEM) Analysis

TEM images were acquired to examine the morphology of the AgNPs. A drop of each nanoparticle suspension was deposited onto a standard copper grid, which was then coated with an amorphous carbon membrane. The TEM analysis was performed using a JEOL JEM-2100 (Tokyo, Japan) microscope at an accelerating voltage of 200 kV. After, images were analyzed using ImageJ V1.53 (U.S. National Institutes of Health, Bethesda, MD, USA) software to assess the mean particle size.

### 2.5. Biological Activity

The experimental conditions selected for the in vitro biological assays are represented in the following Table 1.

#### 2.5.1. In Vitro Cytocompatibility

##### Cell Culture Conditions

The in vitro cytocompatibility of the materials was evaluated in accordance with ISO 10993 standards [35], using commercially available hBM-MC cells. These cells were cultured in alpha minimum essential medium (α-MEM), supplemented with 10% fetal bovine serum (FBS), 100 IU/mL penicillin, 100 µg/mL streptomycin, and 2.5 µg/mL amphotericin B (all reagents sourced from Gibco, Bridgewater, NJ, USA). The cultures were maintained at 37 °C in a humidified atmosphere with 5% CO_2_.

##### Direct Cytocompatibility Assay

This assay evaluated the ability of hBM-MCs to proliferate when cultured directly on the surface of the β-TCP scaffolds. hBM-MCs were seeded onto β-TCP pellets at a density of 3 × 10^5^ cells/cm^2^ and cultured for 12 days in complete culture medium within 24-well plates. The metabolic activity of the hBM-MCs on the β-TCP samples was assessed using the resazurin assay on Days 3, 6, 9, and 12. All samples were transferred to fresh well plates and incubated for 3 h in a 10% resazurin solution (resazurin sodium salt, 0.1 mg/mL, Sigma-Aldrich, St. Louis, MO, USA) prepared in complete medium at 37 °C. Fluorescence (530 nm excitation/590 nm emission) was measured using a microplate reader (Synergy HT, Biotek, Winooski, VT, USA) with Gen5 1.09 Data Analysis Software. The results were plotted relative to the control (β-TCP scaffolds without laser treatment).

### 2.6. Antibacterial Activity

#### 2.6.1. Bacterial Culture Conditions

The antibacterial activity of β-TCP ceramic scaffolds was assessed against *S. aureus* (ATCC 25923) [36]. Bacterial suspensions were prepared by incubating the bacteria in tryptic soy broth (TSB, Liofilchem, Roseto degli Abruzzi, Italy) at 37 °C until reaching a concentration of 10^6^ Colony-Forming Units (CFUs)/mL.

#### 2.6.2. Antibacterial Activity Assay

In this assay, the upper surfaces of the β-TCP scaffolds were exposed to 1 mL of the prepared bacterial suspension in 24-well plates and incubated for 24 h at 37 °C. Antibacterial activity was assessed for both sessile bacteria (attached to the material surface) and the planktonic population (freely floating cells). After incubation, the scaffolds colonized with the sessile cells were transferred to fresh wells, pre-washed with a sterile saline solution (0.9% NaCl), and incubated for 1 h in a 10% resazurin solution prepared in tryptic soy broth (TSB) medium. The same procedure was followed for the planktonic cells remaining in the original wells. Fluorescence was then measured at 530 nm excitation and 590 nm emission using a microplate reader (Synergy HT, Biotek, Winooski, VT, USA) with Gen5 1.09 Data Analysis Software. The results were plotted relative to the control (β-TCP scaffolds without laser treatment).

### 2.7. SEM Characterization of β-TCP Scaffolds Post-Biological Testing

β-TCP samples, either with hBM-MSCs or *S. aureus* adhered, were initially fixed in a 1.5% glutaraldehyde solution prepared in 0.1 M sodium cacodylate buffer (TAAB Laboratories Equipment Ltd., Aldermaston, UK) for 15 min, followed by storage in sodium cacodylate buffer. The samples were then dehydrated through a graded series of ethanol concentrations (50%, 70%, 90%, and 100%) and subjected to critical point drying (CPD 7501, Polaron Range). To enhance imaging quality, the scaffolds were sputter-coated with a thin layer of gold palladium (~4 nm). The morphological features of the β-TCP scaffolds were subsequently analyzed using SEM with a FEI Quanta 400 FEG ESEM/EDAX Genesis X4M (Fei Company, Hillsboro, OR, USA).

### 2.8. Statistical Analysis

Results are presented relative to the untreated samples, which were set as the control group (control = 1.0). All data were analyzed as mean ± standard deviation (SD), and each assay was repeated in triplicate. Statistical significance was determined using one-way ANOVA, with significance levels set at * *p* < 0.05, ** *p* < 0.01, *** *p* < 0.001, and **** *p* < 0.0001. All analyses were performed using GraphPad Prism version 9.0.0 (GraphPad Software, Inc., San Diego, CA, USA).

## 3. Results

### 3.1. Morphological Characterization of the Laser-Treated β-TCP Scaffolds

In this part of the study, the surface properties of fs-laser-treated β-TCP scaffolds were examined using SEM for high-resolution imaging, 3D optical profilometry to assess surface roughness, and a Drop Shape Analyzer to evaluate wettability.

#### 3.1.1. Scanning Electron Microscopy (SEM)

**Figure 3:**
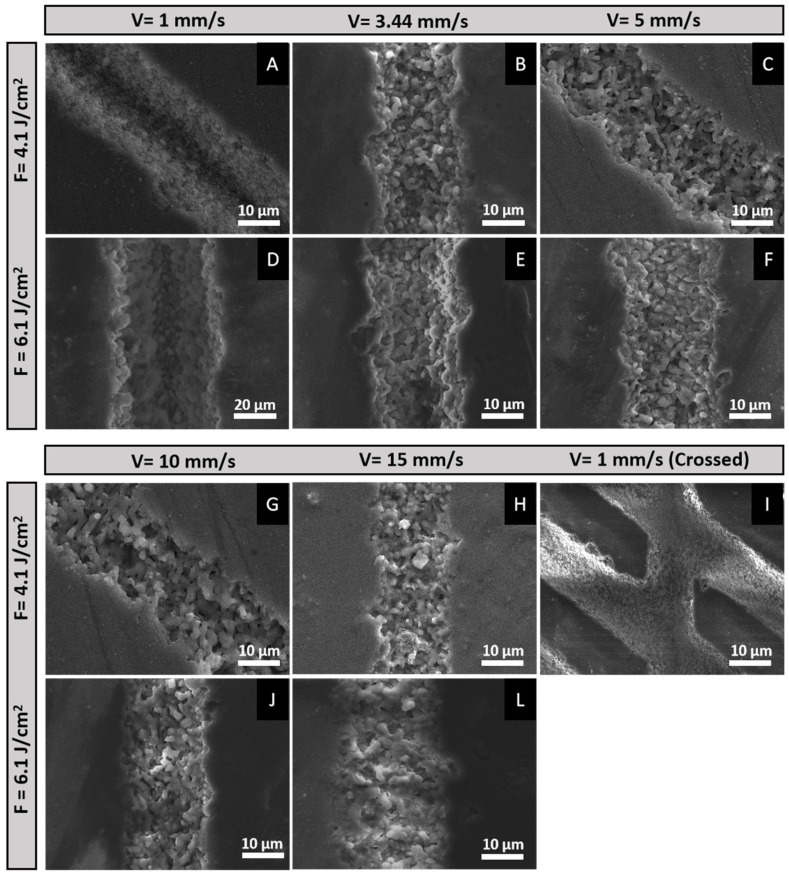
SEM micrographs illustrating the morphological changes in β-TCP samples induced by fs-laser treatment at various fluences (6.1 and 4.1 J/cm^2^), scanning velocities (1, 3.44, 5, 10, and 15 mm/s), and patterns (linear and crossed). Subfigures: (**A**) V = 1 mm/s, F = 4.1 J/cm^2^; (**B**) V = 3.44 mm/s, F = 4.1 J/cm^2^; (**C**) V = 5 mm/s, F = 4.1 J/cm^2^; (**D**) V = 1 mm/s, F = 6.1 J/cm^2^; (**E**) V = 3.44 mm/s, F = 6.1 J/cm^2^; (**F**) V = 5 mm/s, F = 6.1 J/cm^2^; (**G**) V = 10 mm/s, F = 4.1 J/cm^2^; (**H**) V = 15 mm/s, F = 4.1 J/cm^2^; (**I**) V = 1 mm/s (Crossed), F = 4.1 J/cm^2^; (**J**) V = 10 mm/s, F = 6.1 J/cm^2^; (**L**) V = 15 mm/s, F = 6.1 J/cm^2^. All micrographs were acquired with an acceleration of 20 kV and a magnification of 5000×.

The SEM results depicted in Figure 2 clearly demonstrate that the various laser conditions applied have led to distinct physical alterations of the surface of β-TCP samples. One of the key observations is the influence of scanning velocity on the structural organization of the material. For instance, at a scanning velocity of 1 mm/s at both tested fluences, there is a noticeable improvement in structural organization at the nanoscale (Figure 3A). In contrast, increasing the velocity to 3.44 mm/s disrupts this organization, leading to the emergence of nanoscale pores, as shown in Figure 3B. This trend continues as the velocity is further increased to 15 mm/s, where further structural disruptions occur, resulting in the emergence of granular-like structures and additional nanopores (Figure 3H).

To explore the possibility of increasing interconnectivity between the laser-modified lines, a cross-hatched pattern at a 45° angle was tested using a fluence of 4.1 J/cm^2^ while keeping the velocity at 1 mm/s. This patterning produced rhombohedral shapes that maintained a high level of structural organization (Figure 3I).

The impact of laser fluence on the resulting structures is also clearly demonstrated in Figure 3D–F,J,L). Higher energy levels resulted in more intense ablation and a greater disruption of the material, leading to slightly more disorganized structures with less defined boundaries. However, the effect of scanning velocity remained more critical, as increased velocities, particularly above 3.44 mm/s, led to the formation of more porous structures (Figure 3E,F,J,L).

#### 3.1.2. 3D Optical Profilometry

**Figure 4:**
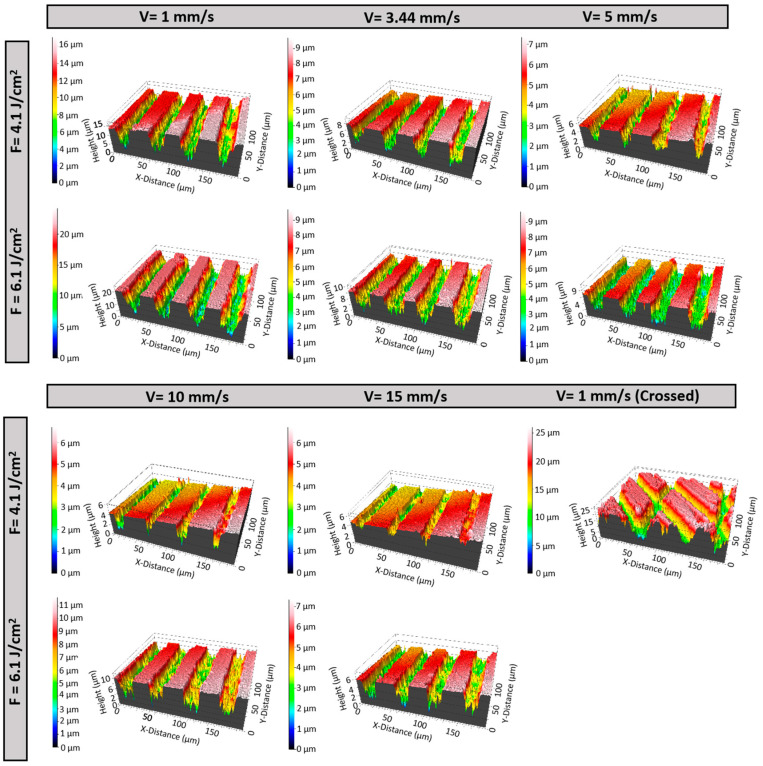
3D optical profilometry images illustrating the groove depth variations in β-TCP samples induced by fs-laser treatment at various fluences (6.1 and 4.1 J/cm^2^), scanning velocities (1, 3.44, 5, 10, and 15 mm/s), and patterns (linear and crossed), acquired with a magnification of 20×.

**Figure 5:**
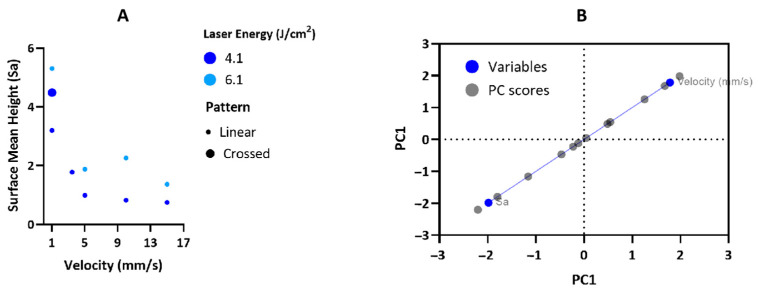
Multivariable bubble plot (**A**) and biplot of PCA analysis (**B**) illustrating the effects of fluence, scanning velocity, and patterns on the surface roughness parameter Sa.

3D optical profilometry revealed significant variations in surface roughness across different laser conditions, as illustrated in Figure 4 and Figure 5. The analysis showed that surface roughness (Sa) varied with both fluence and scanning velocity. At a fluence of 4.1 J/cm^2^, Sa generally decreased as scanning velocity increased, with higher velocities producing smoother surfaces. For example, surfaces treated at 15 mm/s exhibited lower roughness compared to those treated at 5 mm/s under the same fluence (Figure 4 and Figure 5A).

At higher fluences, a different trend was observed. Surface roughness decreased as the scanning velocity increased up to 5 mm/s. However, as the velocity increased further to 10 mm/s, surface roughness began to rise again before decreasing once more at 15 mm/s. This suggests a more complex interaction between laser fluence and velocity at these higher fluences (Figure 4 and Figure 5A).

The biplot in Figure 5B, representing the Principal Component Analysis (PCA), further clarified the strong relationship between Sa and scanning velocity. The PCA analysis revealed a strong inverse correlation at lower velocities, with surface roughness decreasing as scanning velocity increased. However, at higher velocities and fluences, this trend reversed, showing an increase in roughness.

Additionally, the introduction of crossing laser patterns led to a marked increase in Sa across all conditions. The intersecting laser paths generated additional surface features, significantly enhancing roughness compared to conditions without such patterns (Figure 5A).

#### 3.1.3. Wettability Analysis

**Figure 6:**
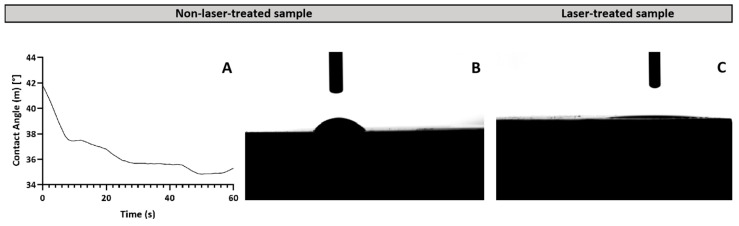
(**A**) Graph illustrating the variation in contact angle over time for non-laser-treated samples, indicating changes in wettability. (**B**) Representative image of a water droplet on a non-laser-treated sample, demonstrating its wettability. (**C**) Comparison image of a water droplet on an fs-laser-treated sample, highlighting the increased wettability of the treated surface.

The impact of laser treatments on surface wettability was assessed by measuring the water contact angles using the sessile drop method. The results showed that laser-induced modifications significantly decreased the water contact angle (WCA) on the treated surfaces (Figure 6C) in relation to the non-laser treated samples (Figure 6A,B). In fact, the WCA was reduced to the point where it became impossible to measure the angle accurately, indicating that the surfaces had transitioned from hydrophilic to superwetting following laser ablation (Figure 6C).

### 3.2. Green-Laser-Assisted Synthesis of AgNPs

In this section of the study, we investigated the synthesis of AgNPs through the photoreduction of silver nitrate (AgNO_3_), utilizing green tea extract as both a stabilizing and reducing agent. The focus was on examining the effects of varying laser fluence levels on the formation and characteristics of the AgNPs.

#### 3.2.1. UV–Vis Spectroscopy

**Figure 7:**
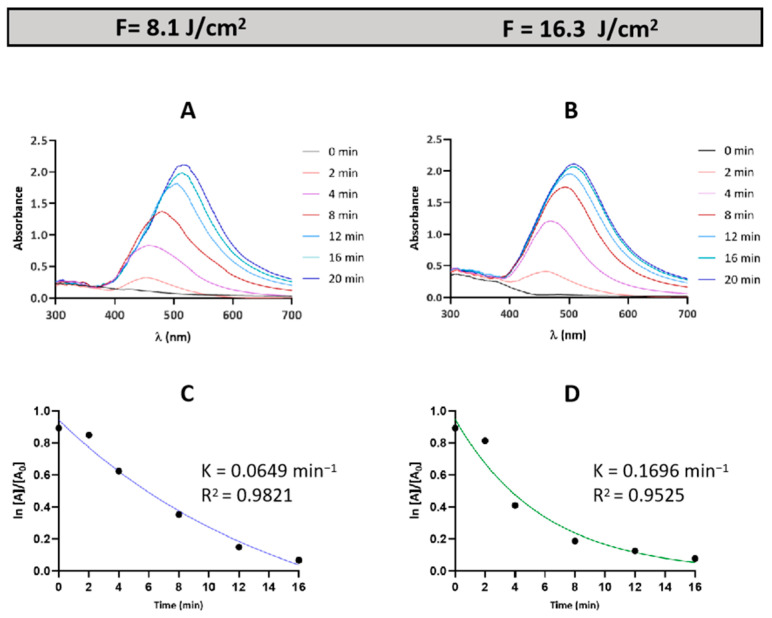
UV–vis spectra showing the SPR peaks of synthesized AgNPs at laser fluences of 8.1 J/cm^2^ (**A**) and 16.3 J/cm^2^ (**B**). Graphs depicting the pseudo-first-order kinetics for the reduction of Ag^+^ ions at fluences of 8.1 J/cm^2^ (**C**) and 16.3 J/cm^2^ (**D**).

The synthesis process of AgNPs was monitored using UV–vis spectroscopy, which revealed a Surface Plasmon Resonance (SPR) peak in the 400–550 nm range, indicative of nanoparticle formation (Figure 7A,B).

After just 2 min of synthesis, the SPR signal became detectable, with a marked increase observed at 8 min and reaching its maximum peak intensity at 20 min. The SPR peak intensity was then employed to analyze the kinetics of nanoparticle formation under two different laser fluence conditions (8.1 and 16.3 J/cm^2^). It was assumed that the formation of AgNPs follows pseudo-first-order kinetics. The concentration of Ag ions at any given time is inversely related to the concentration of AgNPs in the solution, assuming that all Ag ions are converted into AgNPs. Since the concentration of AgNPs correlates directly with the SPR peak intensity, the AgNP formation at a specific time was determined by the following [37]:

(2) $$\left[AgNPs\right]\%=\left(\frac{SPR\;intensity\;at\;time\;“t”}{Maximum\;SPR\;peak}\right)\times 100$$

where *t* denotes the elapsed time. Thus, the concentration of ${Ag}^{+}$ was calculated using

(3) $$\left[{Ag}^{+}\right]\%=100-\left[AgNPs\right]\%\;$$

The natural logarithm of Ag^+^% decreases in a relatively linear manner (Figure 7C,D), confirming the proposed pseudo-first-order reaction kinetics.

The highest rate constant was observed at a fluence of 16.3 J/cm^2^ (Figure 7D), indicating an optimal balance between electron production and the reducing/stabilizing activity of the GTLE. However, under this condition, the data did not exhibit a perfectly linear distribution, although the coefficient of determination (R^2^) (0.9525) was sufficiently high to consider the fit acceptable. In contrast, at the lower fluence, the rate constant was lower but showed a better fit to the model (R^2^ = 0.9821) (Figure 7C).

A key observation was the absence of a UV–vis signal when only silver nitrate and water were used, without green tea extract. This indicates that AgNO_3_ could not be reduced to AgNPs in aqueous solution due to rapid back oxidation by hydroxyl radicals (OH•). The relevant back-oxidation reaction could be described as follows:

(4) $${Ag}^{0}+\mathrm{OH}•\;\to \;{Ag}^{+}+{OH}^{-}$$

In the absence of GTLE, the hydroxyl radicals generated during laser irradiation rapidly oxidize the AgNPs back to silver ions, preventing stable AgNP formation, and thus no SPR signal is observed.

Additionally, it was evident from the SPR peaks (Figure 7A,B) that a red shift occurred over time across all fluence levels, suggesting an increase in nanoparticle size. Larger nanoparticles exhibit SPR at longer wavelengths, indicating growth in nanoparticle size as the reaction progresses [38].

#### 3.2.2. FTIR Analysis

**Figure 8:**
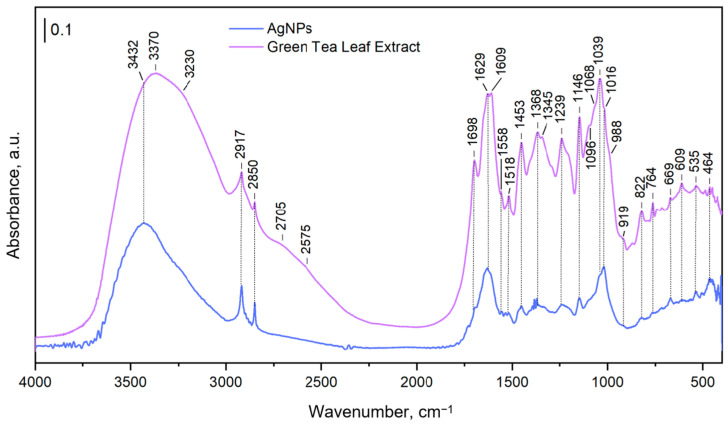
FTIR spectra showing the characteristic vibrational bands of the GTLE and the synthesized AgNPs.

The FTIR spectra depicted in Figure 8 of AgNPs, compared to the GTLE alone, revealed several key peaks that suggest the involvement of specific functional groups in the synthesis process. The broad peak observed at 3432 cm^−1^ in the AgNP spectrum, which shifts from 3370 cm^−1^ in the green tea extract, was assigned to O–H stretching vibrations, indicating the presence of hydroxyl groups. The C=O stretching peak at 1698 cm^−1^ appears in both spectra, suggesting that carbonyl groups are involved. Additionally, aromatic C=C stretching peaks were identified at 1609 cm^−1^ and 1518 cm^−1^, which are characteristic of the aromatic rings found in catechins and other polyphenols. The C–H bending vibration at 1453 cm^−1^ was also noted, along with peaks ranging from 1345 cm^−1^ to 1069 cm^−1^, attributed to C–O–C and C–O stretching, which are commonly found in ether and ester bonds.

#### 3.2.3. TEM Analysis

**Figure 9:**
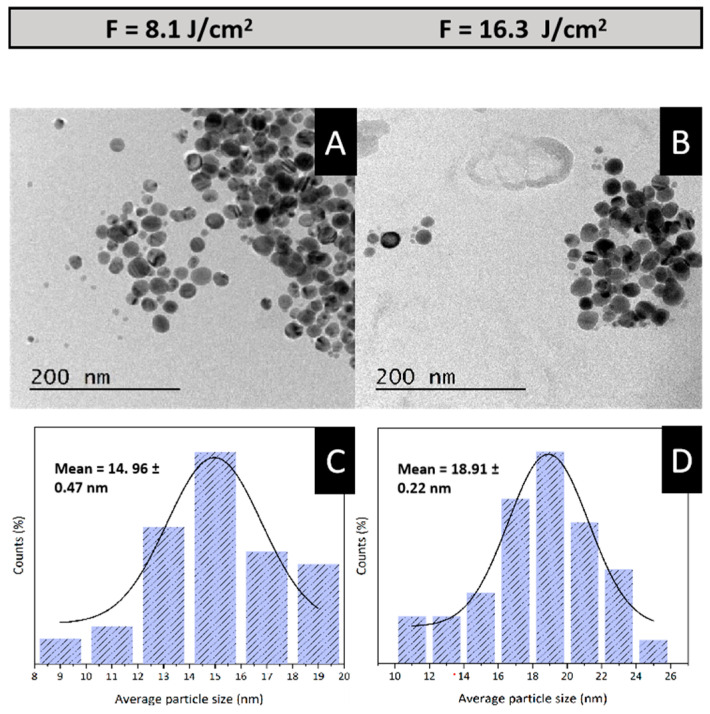
TEM micrographs illustrating the morphology of AgNPs synthesized at fluences of 8.1 J/cm^2^ (**A**) and 16.3 J/cm^2^ (**B**); corresponding histograms showing the size distribution of AgNPs synthesized at these fluences, 8.1 J/cm^2^ (**C**) and 16.3 J/cm^2^ (**D**).

TEM was utilized to assess the morphology and size of the synthesized nanoparticles. As illustrated in Figure 9A,B, the majority of the AgNPs predominantly exhibit a spherical morphology and display a narrow size distribution. Furthermore, the results presented in Figure 9C,D demonstrate that increasing the laser fluence significantly affects nanoparticle size. Specifically, at a laser fluence of 16.3 J/cm^2^, the average nanoparticle size increased to approximately 19 nm, highlighting a clear correlation between laser fluence and particle size.

Considering these findings, the synthesis condition at a fluence of 8.1 J/cm^2^ was selected for further experimentation as it produced the smallest nanoparticles with a favorable size distribution, making it a promising candidate for deposition on β-TCP and subsequent biological testing.

### 3.3. In Vitro Cytocompatibility

In this phase of the study, cytocompatibility was evaluated by examining hBM-MCs growth on fs-laser-treated surfaces, using the resazurin assay to measure cellular metabolic activity. The fs-laser-modified samples included in the tests were those that showed the most promise in terms of nanoscale structural organization: linear pattern (F = 4.1 J/cm^2^), linear pattern (F = 6.1 J/cm^2^), crossed pattern (F = 4.1 J/cm^2^), and linear pattern (F = 4.1 J/cm^2^)@AgNPs.

**Figure 10:**
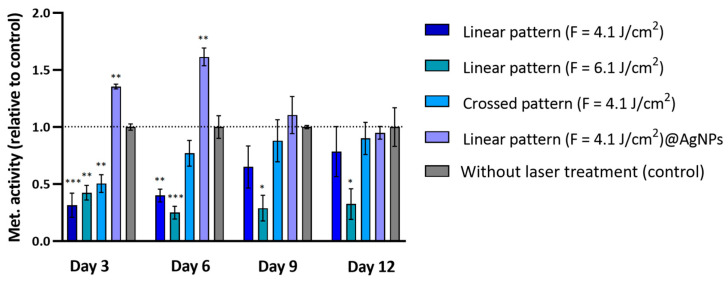
Metabolic activity of hBM-MSCs cultured over the fs-laser-treated β-TCP scaffolds for periods up to 12 days. Results are presented relative to the untreated samples (control, set up at 1.0, dotted line). Statistically different from control: * *p* < 0.05, ** *p* < 0.01 and *** *p* < 0.001.

Figure 10 reveals a nuanced relationship between surface modifications induced by varying laser fluence and the resultant cellular responses. The linear pattern at higher fluence (F = 6.1 J/cm^2^) consistently exhibited lower metabolic activity across all time points. On Day 3, this condition showed a significant reduction in cell viability by approximately 57% (95% CI: 0.1947 to 0.9553, *p* < 0.01), and, afterward, this reduction increased to around 75% (95% CI: 0.4589 to 1.041, *p* < 0.001) (Figure 10).

Conversely, cells on the linear pattern (F = 4.1 J/cm^2^) and the crossed pattern (F = 4.1 J/cm^2^) exhibited a sustained increase in metabolic activity over time despite initially low values. On the linear pattern (F = 4.1 J/cm^2^), cells showed a metabolic activity of about 32% of the control on Day 3 (95% CI: 0.3047 to 1.065, *p* < 0.001); however, this effect gradually diminished, with metabolic activity increasing to approximately 65% of the control by Day 9 and further to 80% by Day 12 (Figure 10). The crossed pattern (F = 4.1 J/cm^2^) followed a similar trend, with initial metabolic activity at around 49% of the control on Day 3 (95% CI: 0.1147 to 0.8753, *p* < 0.01), which became non-significant by Day 6 and continued to increase slightly thereafter (Figure 10).

**Figure 11:**
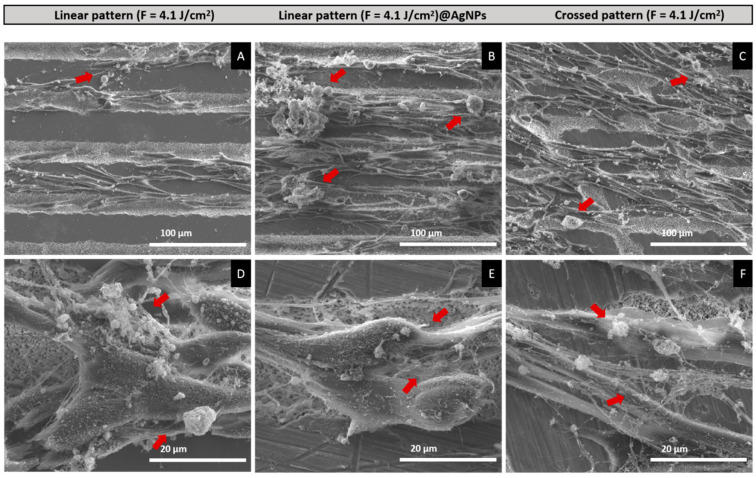
SEM images of human hBM-MSCs cultured over the fs-laser-treated β-TCP scaffolds for 12 days. Low (**A**–**C**) and high (**D**–**F**) magnification images (1000× and 5000×, respectively). Red arrows: examples of mineralized deposits.

The inclusion of AgNPs in the linear pattern (F = 4.1 J/cm^2^) notably enhanced cell viability, especially on Day 6, where a metabolic activity of 162% of control was observed (95% CI: 0.2984 to 0.8350, *p* < 0.01) (Figure 10). Additionally, SEM analysis revealed that cells on these scaffolds: linear pattern (F = 4.1 J/cm^2^), linear pattern (F = 4.1 J/cm^2^)@AgNPs, and crossed pattern (F = 4.1 J/cm^2^) exhibited elongation along the surface patterns (Figure 11A–C) and displayed mineralized matrix formations and vesicular bodies (Figure 11D–F).

### 3.4. Antibacterial Activity

In this section, the antibacterial activity of β-TCP scaffolds against *Staphylococcus aureus* was evaluated. Bacterial suspensions were prepared and incubated to reach a concentration of 10^6^ CFU/mL. The scaffolds were exposed to these suspensions for 6 h, and antibacterial activity was assessed for both sessile and planktonic bacteria using the resazurin assay.

**Figure 12:**
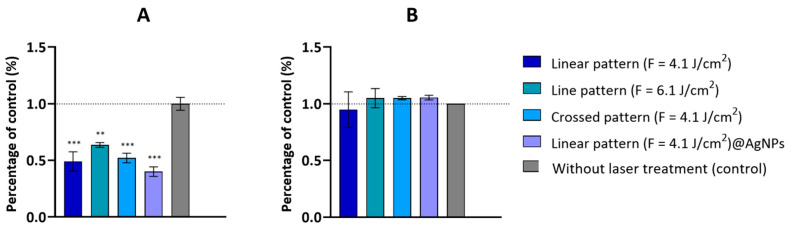
Antibacterial activity of the fs-laser-treated β-TCP scaffolds against sessile (**A**) and planktonic (**B**) growth of *S. aureus*. Results are presented relative to the untreated samples (control, set up at 1.0, dotted line). Statistically different from control: ** *p* < 0.01 and *** *p* < 0.001.

As shown in Figure 12A, fs-laser-treated β-TCP scaffolds significantly reduced the sessile bacterial population. The most pronounced effect was observed with the linear pattern (F = 4.1 J/cm^2^), especially when combined with silver nanoparticles (AgNPs), resulting in a 50% (95% CI: 0.3231 to 0.6969, *p* < 0.001) and 60% (95% CI: 0.4131 to 0.7869, *p* < 0.001) decrease compared to the control values, respectively (Figure 12A). The linear pattern produced with higher values of applied laser fluence (F = 6.1 J/cm^3^) achieved a 36% reduction in bacterial viability (95% CI: 0.1781 to 0.5519, *p* < 0.01) (Figure 12A), while the crossed pattern (F = 4.1 J/cm^2^) resulted in a 48% reduction in bacterial adhesion (95% CI: 0.2931 to 0.6669, *p* < 0.001), although it was less effective than the linear pattern (F = 4.1 J/cm^2^) (Figure 12A).

**Figure 13:**
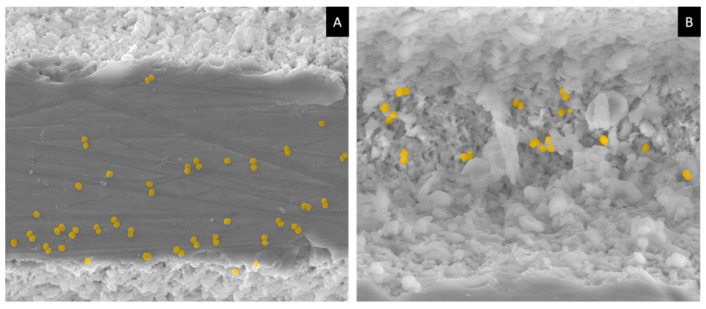
Representative SEM micrographs showing *S. aureus* cells (yellow) adhered to the non-laser-treated region of the β-TCP surface (**A**) and to the bottom of the laser-treated region (**B**).

On the other hand, none of the tested conditions showed a significant antibacterial effect on the planktonic population of *S. aureus* (Figure 12B). This suggests that the observed antibacterial activity is primarily due to the prevention of bacterial adhesion rather than an effect on the surrounding microenvironment of planktonic bacteria. SEM images further supported this, showing bacterial cells on the scaffold surfaces with minimal structural disruption (Figure 13). SEM images also revealed that bacterial accumulation was more pronounced in untreated areas compared to treated regions, underscoring the effectiveness of the laser treatment. In the fs-laser-treated zones, some bacterial populations were observed; however, they were primarily located in the centers of the modified areas, where the surface’s structural organization was less pronounced (Figure 13B).

## 4. Discussion

The femtosecond-laser-processing approach employed in this work has proven to be a versatile tool, not only for structuring β-TCP but also for assisting in the synthesis of AgNPs. This dual functionality significantly impacts bacterial adhesion and the proliferation of hBM-MSCs, making it a promising approach for biomedical applications. SEM morphological analysis shows that at lower scanning velocities (e.g., 1 mm/s), the laser processing has prolonged interaction with the material surface, leading to the formation of self-organized nanostructures due to cumulative energy input. This level of structural organization is particularly notable, as it is challenging to achieve in ceramic materials. Similar phenomena, although rare, have been observed in previous studies, with structures resembling laser-induced periodic surface structures (LIPSSs) [39]. As the scanning velocity increases, the energy deposited per unit area decreases, resulting in less controlled ablation and the formation of more porous, less organized structures. This disruption in structural organization at velocities above 3.44 mm/s is consistent with reduced energy deposition, leading to nanoscale porosity. Such porosity is beneficial for osteointegration [40]; however, it must be balanced with the need to maintain antibacterial properties on the material’s surface. While higher laser energy levels intensify the ablation process, creating slightly disorganized structures, scanning velocity plays a more critical role in determining the final structure. The emergence of increased porosity at higher velocities underscores the importance of careful control over scanning velocity to optimize both the structural integrity and functional properties of the treated material [41].

Three-dimensional optical profilometry reveals a decrease in surface roughness with increasing scanning velocity at 4.1 J/cm^2^. This trend likely arises from reduced energy deposition at higher velocities, resulting in less intense ablation and finer surface texture. However, at higher fluences, the relationship between scanning velocity and surface roughness becomes more complex. Initially, as velocity increases up to 5 mm/s, surface roughness decreases, likely due to more efficient energy distribution and less aggressive ablation [42]. Beyond 5 mm/s, surface roughness initially increases as the velocity reaches 10 mm/s, likely due to insufficient interaction time, leading to incomplete melting or re-solidification and the formation of irregular surface features. However, at the higher velocity of 15 mm/s, the roughness decreases again, possibly due to a more uniform distribution of energy, which promotes smoother re-solidification and reduces the formation of irregularities [43]. PCA suggests that while velocity is a key factor in determining surface roughness, the interplay between velocity and energy density becomes increasingly complex at higher fluences. The increased roughness observed with crossing laser patterns could be due to the complex texturing effects introduced by overlapping laser spots, which enhance surface roughness compared to non-overlapping conditions. Furthermore, the wettability assay shows that laser treatment enhances hydrophilicity, which can be explained by the Wenzel model, where increased surface roughness leads to a larger actual contact area between the surface and liquid. This, in turn, enhances wettability and lowers the contact angle. The transition to superwetting surfaces may have significant implications for biomedical applications, particularly in improving protein adhesion, which could lead to more efficient tissue integration and a better incorporation of implants [44].

In the synthesis of AgNPs, UV–vis spectroscopy reveals that in the GTLE, hydroxyl radicals generated during laser irradiation rapidly oxidize AgNPs back into silver ions, preventing the formation of stable nanoparticles. This results in the absence of a SPR signal, as observed by Tibbetts [45]. GTLE plays a crucial role in this synthesis process. Its polyphenols facilitate the reduction of silver ions to nanoparticles and stabilize them by adsorbing onto their surface [28,46]. Additionally, these polyphenols can scavenge hydroxyl radicals, protecting the nanoparticles from oxidation [47].

The red shift of the SPR peak observed in the UV–vis results contrasts with the blue shift reported in studies using ammonium as a stabilizing agent [48,49]. This discrepancy may be due to the more complex stabilization environment provided by GTLE, which is rich in polyphenols. These not only stabilize the nanoparticles but also promote their growth, leading to a continuous increase in AgNP size and the observed red-shifted SPR peak over time. The increase in the rate constant at higher laser fluences is likely due to the higher density of hydrated electrons generated under these conditions [50]. These electrons act as highly reactive reducing agents, accelerating metal ion reduction and promoting faster nucleation and crystal growth, consistent with the larger nanoparticle sizes observed at higher fluence levels, as revealed by TEM analysis. The TEM results showing that most AgNPs are spherical and small in size are promising, as the literature suggests that such nanoparticles are more susceptible to Ag+ ion release due to their larger surface area, potentially enhancing their beneficial effects [51].

FTIR analysis supports the hypothesis that GTLE polyphenols play a crucial role in the reduction and stabilization of AgNPs. The observed O–H stretching peaks indicate the possible involvement of hydroxyl groups in these processes, while the presence of a C=O stretching peak in both the GTLE and AgNPs suggests that carbonyl groups found in polyphenolic compounds like flavonoids might be involved in capping the AgNPs [52]. Aromatic C=C stretching peaks, along with C-H bending vibrations, imply that catechins in particular could be forming a protective layer around the nanoparticles, aiding in their stabilization [53]. Peaks associated with ether and ester bonds [54] further suggest that esterified polyphenols in GTLE might also contribute to nanoparticle stabilization. These findings support the hypothesis that bioactive compounds in GTLE, including polyphenols, may enhance the stability and biological activity of synthesized AgNPs [55].

Cytocompatibility assays on hBM-MSCs reveal that cells on the linear pattern (F = 6.1 J/cm^3^) exhibited compromised behavior, which may be correlated with increased surface roughness and wettability, potentially leading to the enhanced release of exacerbate phosphate and calcium ions. Elevated cytosolic calcium ion concentrations can be detrimental, potentially leading to cell death [56,57]. However, the linear pattern (F = 4.1 J/cm^2^) and the crossed pattern (F = 4.1 J/cm^2^) demonstrated improved cell viability over time despite initial cytotoxic effects. This suggests that while these conditions initially stress the cells, they do not induce long-term adverse effects; instead, the cells adapt to the modified surfaces, gradually increasing their viability. The significant enhancement in cell viability observed with the inclusion of AgNPs in the linear pattern (F = 4.1 J/cm^2^), particularly on Day 6, may be attributed to the synergistic effects of AgNPs and the potential presence of phenolic compounds adhered to their surface. The antioxidant and anti-inflammatory properties of these phenolic groups likely mitigate oxidative stress induced by the scaffold surface modifications and initial ion release, thereby creating a more favorable environment for cell growth [58]. However, a decrease in cell viability/proliferation was observed after Day 6, potentially due to the high initial stimulation of cell growth by AgNPs, which may have led to the earlier confluence of the cell layer and subsequent contact inhibition, resulting in the abrupt arrest of the cell cycle typically seen in rapidly proliferating cultures [59,60]. This is supported by the observation that the viability values on Days 9 and 12 were comparable to those in the samples with the linear pattern (F = 4.1 J/cm^3^), crossed pattern (F = 4.1 J/cm^3^), and untreated. Therefore, an intrinsic cytotoxic effect of the AgNPs is ruled out. Another factor that may explain the observed reduction in viability is the potential differentiation of the cells. A metabolomic study conducted by Bispo et al. [61] identified significant changes in over 30 metabolites during the 21-day osteogenic differentiation period, highlighting the dynamic nature of this process. Variations in metabolic activity during differentiation could be influencing the viability results obtained in the resazurin assay. SEM results further support this notion, showing that cells on the scaffolds not only exhibited elongation along the surface patterns but also displayed mineralized matrix formations and vesicular bodies, suggesting early signs of bone tissue differentiation and bone matrix formation [62].

Conversely, antibacterial assays revealed a significant reduction in sessile bacteria on laser-treated β-TCP scaffolds, particularly those with the linear pattern (F = 4.1 J/cm^2^) combined with AgNPs. This effect is likely related to the creation of nanoscale self-organized features resembling LIPSS, which may prevent bacterial colonization [63], as well as the synergistic antibacterial or anti-adhesive effects of AgNPs. The primary mechanism appears to be the creation of an inhospitable surface environment rather than direct bactericidal activity. Higher fluence treatments and crossed patterns also reduce bacterial adhesion but are less effective compared to the linear pattern with AgNPs, suggesting that AgNPs are critical in preventing bacterial attachment. The lack of antibacterial effects on free-floating bacteria in the planktonic cell assay supports the hypothesis that the antibacterial activity observed is more likely due to surface modifications and AgNP doping, resulting in an anti-adhesive surface. Beyond the direct effects of structures obtained by laser ablation, a quantitative proteomic study by Zhang et al. suggested the involvement of AgNPs and silver ions in chemotaxis [64]. Additionally, Li et al. observed the inhibition of several enzymes and ATP-binding proteins in *S. aureus* [65], and Mirzajani et al. reported the inhibition of several membrane proteins involved in the electron transport system of *Bacillus thuringiensis* [66]. We hypothesize that AgNPs do not necessarily need to internalize into bacterial cells to prevent their adhesion; instead, they may disrupt chemotactic signaling pathways or trigger metabolic deregulation through interaction with the bacterial membrane. However, further analysis is required to fully understand these mechanisms.

To evaluate the proposed hypotheses of this study, a specific null hypothesis was established. This null hypothesis posits that the use of fs laser for both inducing surface modifications on β-TCP and assisting in the synthesis of AgNPs using GTLE as a stabilizing/reducing agent does not enhance cellular viability while concurrently reducing bacterial viability. This hypothesis was rejected based on significant findings. SEM and 3D optical profilometry analyses revealed that fs-laser processing significantly improved the surface texture and roughness of β-TCP, contributing to increased wettability, which is essential for enhancing bone cell attachment and proliferation. Additionally, the fs laser effectively facilitated the photoreduction synthesis of AgNPs, yielding smaller and spherical nanoparticles. Furthermore, the combination of fs-laser treatment and AgNP incorporation resulted in increased cellular viability and proliferation, along with a substantial reduction in bacterial viability on laser-treated scaffolds. This indicates a synergistic effect, where the enhanced surface properties of the β-TCP, combined with the antimicrobial properties of the synthesized AgNPs, significantly improve cellular viability while effectively reducing bacterial viability.

While these results are promising, further research is essential to translate these findings into clinical applications. Our group is currently investigating the chemical modifications induced by laser treatment on β-TCP and how these changes influence ionic release and interactions with AgNPs, which could impact long-term implant stability and antibacterial efficacy. Understanding how laser-induced surface patterns affect cell differentiation and molecular interactions with bacteria is also critical, as these factors are key to optimizing scaffolds for bone regeneration and infection prevention. Despite the potential of this technology, challenges remain. The scalability of femtosecond laser processing must be carefully evaluated for clinical use, particularly in terms of cost and operational complexity. Additionally, the long-term stability and biological effects of AgNPs need in vivo validation to ensure their safety and effectiveness in medical devices. Future studies should refine laser parameters to further enhance structural and functional properties, with a focus on how these surfaces interact with biological systems at a molecular level. Comprehensive in vivo assessments of the biocompatibility and performance of laser-modified scaffolds will be crucial to their successful integration in clinical settings, particularly in tissue engineering and implantable medical devices.

## 5. Conclusions

This study underscores the translational potential of fs-laser treatment as a transformative approach for enhancing the clinical performance of β-TCP-based biomaterials. By precisely modifying surface characteristics—creating organized nanostructures through controlled scanning velocities that effectively guide hBM-MSCs while repelling bacterial colonization—this method significantly improves osteointegration, which is crucial for successful bone regeneration and long-term implant stability. Additionally, the integration of GTLE polyphenols in the synthesis of AgNPs has demonstrated substantial promise, yielding small, spherical nanoparticles that not only enhance cell viability but also reduce bacterial adhesion on the treated surfaces. These synergistic effects illustrate the potential of laser-assisted surface engineering to develop advanced medical implants with superior tissue compatibility and antibacterial properties. To fully realize these benefits in clinical settings, further research is warranted to elucidate the underlying mechanisms of these enhancements and optimize this approach for safe and effective application in bone-regenerative therapies and infection prevention.

## Figures and Tables

**Table 1:** Summary of the parameters used for developing β-TCP samples used for in vitro biological assays.

Scanning Velocity (mm/s)	Fluence (J/cm^2^)	Pattern	Doping
NA	NA	NA	NA
1	4.1	Linear	NA
1	4.1	Linear	AgNPs
1	6.1	Linear	NA
1	4.1	Crossed	NA

## Data Availability

The original contributions presented in the study are included in the article, further inquiries can be directed to the corresponding authors.
